# Prevalence of mental health issues amongst slovak and international medical students at university of pavol jozef Šafárik: A cross-sectional study

**DOI:** 10.1192/j.eurpsy.2021.992

**Published:** 2021-08-13

**Authors:** J. Dragasek, M. Seabrook

**Affiliations:** 1 1st Dept. Of Psychiatry, University of P. J. Safarik, Kosice, Slovak Republic; 2 Faculty Of Medicine, University of P.J. Safarik, Kosice, Slovak Republic

**Keywords:** mental health, Depression, Anxiety, hedonistic capacity

## Abstract

**Introduction:**

The prevalence of mental health issues amongst domestic and foreign students in Slovakian medical schools and any differences between them is currently unknown.

**Objectives:**

The goals of this paper are to determine the prevalence and extent of mental health issues among medical students at Pavol Jozef Šafárik University (UPJŠ) in Kosice, Slovakia and to determine if there is a difference between domestic and foreign students’ mental health at UPJŠ.

**Methods:**

A combined questionnaire utilizing well-known sources was distributed to UPJŠ medical students to self-assess their levels of anxiety, depression and hedonic capacity (Zung, 1965; Zung, 1971; Snaith et al., 1995). Two-tailed T-tests and regression statistical analyses were applied to determine the significance of the data and any differences.

**Results:**

27% (n=319) and 25% (n=300) responses were collected from domestic and foreign UPJŠ medical students, respectively. 57% of domestic and 74% of foreign students screened positive for either anxiety, depression, or a combination. The 17% increased rate of anxiety and/or depression amongst foreign students when compared to domestic students was statistically significant (P<0.001). The differences between the two groups regarding hedonic tone were not statistically significant.

**Conclusions:**

The prevalence of mental health issues amongst domestic and foreign UPJŠ medical students is much higher than the worldwide average. The higher rate of anxiety and depression observed in foreign UPJŠ medical students when compared to domestic students may be due to a reduced social support system as well as studying in a foreign country. These data suggest special support may be necessary for medical students studying abroad.

